# Physicochemical Properties of Starch from High-Quality Hybrid *Indica* Rice: Insights from National High-Quality Rice Gold Award Chinese Varieties

**DOI:** 10.3390/foods15081335

**Published:** 2026-04-11

**Authors:** Yumei Wang, Jiale Wu, Xingeng Wu, Yanhua Zeng, Yongjun Zeng, Feiyu Tang, Xiaobing Xie

**Affiliations:** 1 Key Laboratory of Crop Physiology, Ecology and Genetic Breeding, Ministry of Education, Nanchang 330045, China; ymwang1212@163.com (Y.W.); wujiale0728@stu.jxau.edu.cn (J.W.); 15070208549@163.com (X.W.); zyh74049501@163.com (Y.Z.); zengyj2002@163.com (Y.Z.); fytangcau@163.com (F.T.); 2 School of Agricultural Sciences, Jiangxi Agricultural University, Nanchang 330045, China

**Keywords:** high-quality *indica* rice, amylopectin fine structure, taste-related attributes, functional properties

## Abstract

The physicochemical properties of starch in high-quality hybrid *indica* rice (HQR) varieties that have received the National High-Quality Rice Gold Award are not well characterized. Ten HQR and two ordinary-quality *indica* rice (OQR) varieties were selected for this study. All varieties were identically cultivated under late-season conditions in southern China and were subsequently analyzed for differences in taste-related attributes, amylopectin fine structure, and functional properties. Compared with OQR varieties, HQR varieties exhibited a distinct starch profile: lower amylose (16.6–20.2%) but higher amylopectin content (62.6–65.0%), a greater proportion of small and medium starch granules, and a higher ratio of A and B1 chains in amylopectin (with few exceptions). Functionally, HQR varieties showed significantly (*p* < 0.05) higher gel consistency, solubility, and swelling power, along with higher breakdown but lower setback. They also generally exhibited higher crystallinity and gelatinization enthalpy, alongside a softer texture. Notably, the functional properties showed strong correlations (*p* < 0.05) with most taste-related attributes and amylopectin fine structures across all varieties. These findings provide critical guidance for future breeding programs aimed at improving the quality of *indica* rice and developing new elite HQR varieties.

## 1. Introduction

Rice (*Oryza sativa* L.) is recognized as a crucial cereal crop worldwide, acting as a primary food source for nearly 65% of China’s population. Starch, which constitutes about 90% of the dry weight of milled rice, fundamentally determines the physicochemical and cooking properties of rice grains [1]. These critical properties encompass starch granule size and distribution, the fine structure of amylopectin, pasting profiles, thermal transition parameters, and textural attributes, all of which directly influence eating and cooking quality [2]. Rice starch is mainly composed of two types of glucose polymers: amylose and amylopectin. While amylose predominantly exists in the amorphous areas of the starch granules and forms single helices, amylopectin, which forms double helices, is primarily found in the crystalline regions [3]. Amylose content (AC) is a well-established key factor influencing rice texture and eating quality, with lower levels generally associated with a softer, stickier, and glossier cooked product, while higher levels tend to result in firmer, drier, and more separated grains [4]. To meet consumers’ preference for a soft and sticky texture, rice breeders have focused on selecting low-amylose *indica* rice varieties during varietal selection, resulting in a declining trend in the amylose content of rice globally [5,6,7]. In China, the AC of *indica* rice has dropped from roughly 24% in the 1990s to nearly 17% today [5]. The predominant high-quality varieties of *indica* rice typically show low to intermediate AC levels, which range between 15% and 20% [8].

In addition to AC, the detailed structure of amylopectin, especially the pattern of branch chain lengths, is acknowledged as a critical element influencing functional characteristics such as pasting behavior, gelatinization, and the final texture of the product [9]. The distribution of chain lengths within amylopectin is classified into categories A (DP 6–12), B1 (DP 13–24), B2 (DP 25–36), and B3 (DP > 37) [10]. Research has shown that an increased ratio of shorter chains (A) correlates with lower gelatinization temperatures, enhanced breakdown, and a softer, stickier texture in cooked rice [2,11]. Conversely, a higher proportion of long chains (e.g., B3) contributes to higher gelatinization temperatures, greater paste stability, and a firmer texture [11]. Moreover, the molecular structure of amylopectin plays a crucial role in the arrangement of double helices within crystalline lamellae, significantly affecting starch solubility, swelling capacity, and digestibility [10,12]. Furthermore, the distribution of chain lengths in amylopectin constitutes a vital intrinsic factor that impacts starch granule size [13]. Generally, a larger ratio of shorter chains tends to correlate with a higher quantity of small to medium-sized granules, a critical structural feature of high-quality rice known for its excellent eating properties [14].

*Indica* rice, adapted to tropical and subtropical climates, represents the predominant rice type cultivated in southern China and across much of Asia. Over recent decades, substantial efforts in rice breeding have focused on enhancing yield potential to satisfy growing food demands in China [15]. To this end, the strategic exploitation of heterosis has been instrumental in developing high-yielding hybrid *indica* rice varieties [16]. However, many such hybrid *indica* rice varieties often exhibited poor grain quality, especially in terms of eating and cooking properties, as breeding efforts historically prioritized yield gains over quality traits [17]. With rapid economic development and rising living standards, consumer preferences in China have shifted markedly toward high-quality rice. Consequently, numerous hybrid *indica* rice varieties that combine high yield with enhanced quality have been developed and released in recent years [7].

The National High-Quality Rice Gold Award is presented at the National Rice Taste Quality Evaluation event, which is co-organized by the National Agricultural Technology Extension Service Center and the National Rice Breeding Collaborative Research Group in China. Each year, only ten to fifteen gold award varieties are selected for *indica* and *japonica* rice, respectively. However, the common physicochemical characteristics and fine structural attributes of starch in these improved high-quality *indica* rice (HQR) varieties, as well as how they differ from those of ordinary-quality *indica* rice (OQR), remain insufficiently understood. In particular, the multiscale structural and physicochemical characteristics of starch in the HQR varieties recognized by the “National High-Quality Rice Gold Award” have not been systematically characterized. Such knowledge is essential for breeders aiming to further enhance grain quality through targeted selection. We hypothesize that, compared with OQR varieties, HQR varieties will exhibit the following characteristics: in terms of starch composition and granule structure, they will have lower amylose content but higher amylopectin content, with a higher proportion of short chains and a lower proportion of long chains in amylopectin. This results in a greater number of small starch granules and fewer large granules, thereby affecting functional properties. Consequently, HQR varieties will demonstrate improved pasting properties, thermal properties, and textural properties, making the cooked rice softer in texture and less prone to retrogradation.

Therefore, twelve hybrid *indica* rice varieties were selected in this study, including ten HQR varieties that received the National High-Quality Rice Gold Award from the National Rice Taste Quality Evaluation event, and two OQR varieties. These varieties were planted uniformly during the late growing season (from June to November). The objectives were to: (1) investigate and compare the starch compositional traits, granule characteristics, crystalline structure, and amylopectin chain length distribution between HQR and OQR varieties; and (2) elucidate how these structural differences influence the pasting, thermal, and textural properties of starch, thereby providing a scientific basis for the genetic improvement of cooking and eating quality in hybrid *indica* rice.

## 2. Materials and Methods

### 2.1. Plant Materials and Experimental Design

Ten HQR varieties, along with two OQR ones, were selected as experimental materials. The HQR varieties were Tliangyou 131 (TLY131), Longjingyou 2 hao (LJY2), Taifengyou 208 (TFY208), Taiyou 553 (TY553), Taoyouxiangzhan (TYXZ), Wanxiangyou 982 (WXY982), Wanxiangyoushuangzhan (WXYSZ), Yexiangyouhaisi (YXYHS), and Yexiangyoulisi (YXYLS). They received the National High-Quality Rice Gold Award from the National Rice Taste Quality Evaluation event. The OQR varieties were JiyouT025 (JYT025) and Jiyouhang1573 (JYH1573). Detailed information on these tested varieties is provided in Table 1. Field experiments were conducted at the Shanggao Rice Science and Technology Institute of Jiangxi Agricultural University (114°97′ E, 28°23′ N, 93.4 m altitude), Zengjia Village, Sihxi Town, Shanggao County, Jiangxi Province. Seeds were sown on June 26, and seedlings were transplanted on July 17. The trial followed a randomized block design with three replications, and each plot measured 15 m^2^ (3 m × 5 m). The planting, fertilization, irrigation, and pest control practices were identical across all varieties. After harvest, the rice was stored under natural conditions (temperature around 20 °C, humidity 60%) for three months before evaluation of its starch physicochemical properties.

### 2.2. Starch Isolation

The extraction of starch from milled rice was performed using a modified method based on Xiong et al. [18]. In summary, 100 g of milled rice were immersed in a 0.1 mol L^−1^ NaCl solution at ambient temperature for 24 h. Following the soaking period, the softened rice grains were blended to create a slurry. This slurry was then filtered using a 200-mesh sieve, and the filtrate was gathered into 50 mL centrifuge tubes. The samples were subsequently centrifuged at 4000 rpm for 20 min to eliminate impurities. The supernatant, which excluded the non-white upper layer, was carefully discarded, while the white starch pellet beneath was resuspended in distilled water. The resulting suspension was agitated for 20 min and centrifuged once more. This washing procedure was performed five times. The purified starch was then dried at 35 °C for 48 h and finally passed through a 200-mesh sieve.

### 2.3. Starch Components and Protein Content

The amylose content of rice was determined using the iodine-blue colorimetric method in accordance with the Chinese National Standard GB/T 15683-2008 [19]. Total starch content was measured via enzymatic hydrolysis following GB 5009.9-2016 [20]. Amylopectin content was calculated as the difference between total starch and amylose content. The crude protein content was determined following ISO 20483:2013 [21] using an automatic Kjeldahl nitrogen analyzer (Kjeltec 8400, FOSS, Hillerød, Denmark) with a protein conversion factor of 5.95. 

### 2.4. Starch Amylopectin Fine Structure

The fine structure of amylopectin was analyzed using high-performance anion-exchange chromatography with pulsed amperometric detection (HPAEC-PAD) on a Dionex ICS-5000 system (Thermo Fisher Scientific, Sunnyvale, CA, USA), in accordance with the methodology outlined by Xiong et al. [18]. A suspension of starch (10 mg) in water (5 mL) was subjected to heating in a boiling water bath for 1 h. Following this, sodium azide, acetate buffer, and isoamylase were added, and the mixture was incubated for 24 h at 37 °C to facilitate debranching. The resultant glucans underwent treatment with 0.5% (*w*/*v*) sodium borohydride in alkaline conditions for a duration of 20 h. A 600 μL aliquot of the solution was vacuum-dried at room temperature, then dissolved in 30 μL of 1 M NaOH for 60 min, subsequently diluted with 570 μL of distilled water. The prepared samples were subsequently subjected to analysis via HPAEC-PAD.

### 2.5. Scanning Electron Microscopy

The surface morphological characteristics of the isolated starches were examined using a scanning electron microscope (HITACHI Regulus 8100, Tokyo, Japan). The starch samples were suspended in absolute ethanol and then dispersed onto circular metal stubs affixed with double-sided adhesive carbon tape. Subsequently, the samples were coated with gold using a sputter coater to enhance conductivity prior to imaging.

### 2.6. Starch Particle Size Distribution

The distribution of starch granule sizes was analyzed employing a laser diffraction particle size analyzer (Mastersizer 3000, Malvern Instruments, Malvern, UK). In summary, 100 mg of starch was mixed with 1 mL of ultrapure water and stirred at 2000 rpm until thoroughly homogenized. Subsequently, the mixture was placed into the analyzer for measurement. The size distribution of the granules was reported on a volumetric basis.

### 2.7. X-Ray Diffraction (XRD) Pattern

The starch samples’ X-ray diffraction (XRD) patterns were recorded with the use of an X’Pert Pro X-ray diffractometer (PANalytical, Almelo, The Netherlands). These measurements were performed at operating settings of 40 kV and 40 mA, with the diffraction angle (2θ) spanning from 4° to 60° at a scan rate of 4°/min. The percentage of relative crystallinity was calculated by comparing the crystalline region to the overall diffraction area, utilizing MDI Jade 5.0 software. To guarantee reproducibility, each sample underwent three measurements.

### 2.8. Gel Consistency, Swelling Power, and Water Solubility of Starch

Gel consistency was measured according to the method described by Huang et al. [22]. The determination of swelling power and water solubility adhered to a method outlined previously [23], with minor alterations. Initially, the starch sample (M0) was weighed and transferred into a 2 mL centrifuge tube (M1). Subsequently, ten milliliters of distilled water were introduced, and the resulting mixture was subjected to heating in a shaking water bath at 85 °C for 30 min. After cooling to room temperature, the sample underwent centrifugation at 4000× *g* for 15 min, after which the supernatant was discarded. The colloid that remained in the tube (M2) was then weighed, followed by drying the precipitate at 105 °C until a constant weight (M3) was achieved. The calculations for swelling power and solubility were carried out as follows: swelling power = (M2 − M1)/(M3 − M1) (g/g); water solubility (%) = 100 × (M0 + M1 − M3)/M0 × 100%.

### 2.9. Thermal Properties of Starch (DSC)

The thermal properties of starch were analyzed using a differential scanning calorimeter (DSC 200 F3, NETZSCH, Selb, Germany). Data were processed with the Thermal Analysis software (Proteus® version 6.0). Approximately 10 mg of starch was mixed with 30 μL of ultra-pure water in an aluminum crucible, which was then hermetically sealed and equilibrated at room temperature for 24 h. Prior to measurement, the instrument was calibrated with an empty aluminum pan as reference. The sample was heated from 30 °C to 120 °C at a heating rate of 10 °C/min under a nitrogen atmosphere.

### 2.10. Rice Pasting Properties

The characteristics related to the pasting of the starch samples were assessed with a Rapid Visco-Analyzer (RVA Super 1633, Newport Scientific, Warriewood, Australia). In brief, weigh 3.0 g of the starch sample and mix it with 25 mL of distilled water in an RVA aluminum canister. Subsequently, load the canister onto the instrument for testing. The sample is initially heated to 50 °C for 1 min, followed by a temperature increase to 95 °C at a rate of 12 °C/min. The sample is then maintained at 95 °C for 2.5 min before being cooled to 50 °C at the same rate of 12 °C/min and finally held at 50 °C for 2 min. Additionally, the TCW 3 (Thermal Cycle for Windows) software was employed to document the subsequent parameters: Peak viscosity, Hot viscosity, Breakdown, Final viscosity, and Setback.

### 2.11. Rice Textural Properties

The textural properties were evaluated using a texture analyzer (TVT 6700, Perten, Stockholm, Sweden) equipped with a 36 mm cylindrical probe, following the two-cycle compression program described by Xiong et al. [24]. The specific parameters were compression ratio of 80%, test speed of 1.0 mm/s, retraction speed of 2.0 mm/s, trigger force of 5 g, and data collection frequency of 333 pps. Precisely 10 g of milled rice was measured and cooked for 30 min. For each assessment, three intact cooked rice grains were selected, and the measurement was repeated ten times. Textural properties were analyzed using TexCalc 5 software. After omitting the highest and lowest values, the mean of the remaining eight replicates was calculated and reported as the final results for parameters such as firmness, stickiness, springiness, cohesiveness, and chewiness.

### 2.12. Statistical Analysis

Data analysis was performed using Statistix 8.0 (Analytical Software, Tallahassee, FL, USA) to compute mean values and standard deviations. Comparisons among treatments were conducted using the least significant difference (LSD) test at a significance level of *p* < 0.05. Figures were generated using Origin 2021 (Microcal Software, Inc., Northampton, MA, USA). In addition, Pearson correlation analysis was used to examine the correlations between the textural properties of cooked rice and the starch components, structures, and physicochemical properties across all varieties, with significance levels set at 0.05 and 0.01.

## 3. Results and Discussion

### 3.1. Starch Components

Significant differences in grain amylose and amylopectin content among rice varieties were observed (Table 2). HQR varieties had significantly lower amylose content (ranging from 16.6% to 20.2%) than OQR varieties (ranging from 22.6% to 26.1%), whereas they showed significantly higher amylopectin content (ranging from 62.6% to 65.0%) than OQR varieties (ranging from 56.4% to 59.6%). To meet consumer preferences for a soft and glutinous texture, previous studies have indicated that rice breeders focus on selecting *indica* varieties with low amylose content [25]. The synthesis of rice amylose is primarily controlled by granule-bound starch synthase (GBSSI), which is encoded by the Waxy gene. High-quality rice varieties typically carry specific Waxy alleles (e.g., Wx^b, Wx^mq, wx), leading to reduced GBSSI enzyme activity or lower expression levels. As a result, these alleles decrease amylose synthesis and improve rice quality [26,27]. However, there was no consistent trend in protein content between HQR and OQR varieties. Amylose and protein content are widely recognized as the two most critical factors determining rice eating quality [2,13]. Previous studies indicate that high-eating-quality *indica* rice varieties typically exhibit optimal amylose content (13.2–20.6%) and appropriate protein content (5.4–8.4%) [6,8]. Notably, the amylose and protein content of HQR varieties in this study fall within these established ranges. Although both amylopectin and total starch content exhibit relatively weak correlations with eating quality, the molecular structure of amylopectin is closely associated with it [28].

### 3.2. Starch Granule Morphology and Granule Size Distribution

**Figure 1:**
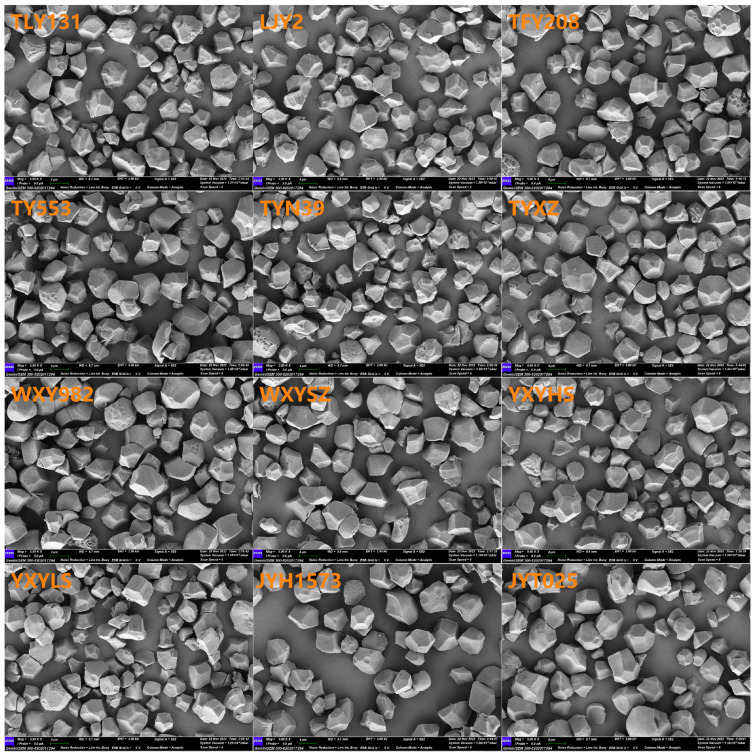
SEM micrographs of starches of HQR and OQR varieties.

The morphological characteristics of starch granules are determined by botanical origin, genetic and environmental factors [1]. In the present study, the starch granules in HQR and OQR varieties exhibited a uniform spherical polyhedral morphology, characterized by distinct edges and sharp angles (Figure 1). However, significant differences were observed in starch granule size distribution and mean diameter across varieties (Table 3). On average, HQR varieties showed significant higher proportion of small granules (<3 μm) and medium granules (3–10 μm) but lower proportion of large granules (>10 μm) as well as smaller mean diameter of volume and surface area than OQR varieties. Furthermore, considerable variations were also observed in granule size distribution among HQR varieties, the proportion of small granules ranged from 7.1% to 12.0%, medium granules from 53.4% to 65.1%, and large granules from 24.1% to 39.5%, as well as the mean diameter of volume which ranged from 9.8–21.5 μm. In general, HQR varieties exhibit a greater proportion of small and medium granules (<10 μm) and smaller mean granules diameter than OQR varieties [29,30]. Certainly, some HQR cultivars also showed a higher or comparable cumulative distribution of large-diameter (>10 μm) granules and larger mean granules diameter in this study (like TFY208) and previous studies [31]. Studies have shown that the smaller the starch granules, the larger their specific surface area, and thus the greater the opportunity for contact with water molecules [32,33]. This leads to faster water absorption and swelling during the early stage of heating, which explains why the HQR variety exhibits higher swelling power. After intense swelling, the structure of the small granules becomes more fragile, making them more susceptible to rupture under continuous heating and shear forces. This rupture results in the leakage of internal materials, thereby reducing the final viscosity, a finding that is entirely consistent with the observation that the HQR variety has a lower final viscosity. In contrast, larger starch granules, after gelatinization, are able to form a more robust and compact gel network, leading to higher rice hardness [34]. This accounts for the greater hardness observed in the OQR variety.

### 3.3. X-Ray Diffraction (XRD) Analysis of Starch Granules

**Figure 2:**
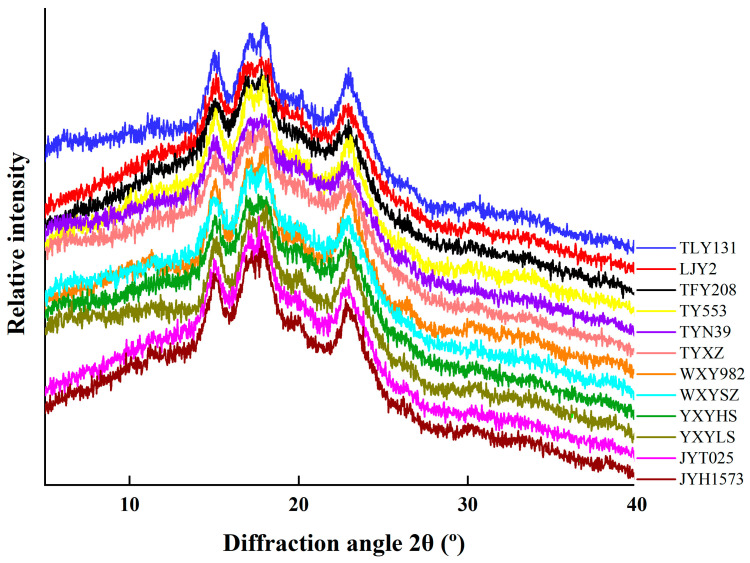
X-ray diffraction (XRD) of HQR and OQR varieties.

The X-ray diffractograms of starches from HQR and OQR varieties are shown in Figure 2. All samples from both types exhibited a similar set of diffraction peaks, including a single peak at approximately 15–16° (2θ), a doublet at 17–18° (2θ), a less intense peak near 20° (2θ), and a broad peak centered around 22–23° (2θ). The diffraction patterns obtained here confirm that all analyzed starches exhibited an A-type crystalline structure, which is consistent with previous reports [33]. In this study, the relative crystallinity of starches of HQR varieties ranged from 24.5% to 32.8%, while OQR varieties ranged from 25.1% to 25.8% (Table 3). Six HQR varieties showed significantly higher relative crystallinity than OQR varieties. Generally, the crystallinity of rice starch is governed by a combination of intrinsic factors, such as amylose content, chain length distribution of amylopectin and starch granule morphology and size, and extrinsic factors, including genetic background, environmental conditions, and cultivation managements [35,36]. The crystalline regions in starch granules are primarily formed by the double-helical packing of short- (DP 6–12) and medium-length (DP 13–24) branched chains of amylopectin, while the linear amylose in amorphous regions disrupts this ordering through steric hindrance and a dilution effect [37,38]. Obviously, some HQR varieties, like TFY208, TY553 and TWXY982, have lower relative crystallinity, which may be due to a smaller proportion of small and medium granules (<10 μm) and greater proportion of large granules.

### 3.4. Amylopectin Fine Structure of Starch

Significant differences in the chain length distribution of amylopectin were observed between HQR and OQR varieties (Table 4). With the exception of TFY208, WXY982 and WXYSZ, HQR varieties showed 10.0–14.8% higher proportion of A (DP 6–12) and 5.0–6.4% lower proportion of B1 (DP 13–24) compared to OQR varieties. In contrast, TFY208, WXY982 and WXYSZ had slightly or significantly lower proportion of A chain and higher proportion of B1 chain than OQR varieties. Additionally, most HQR varieties displayed higher B2 (DP 25–36) but lower B3 (DP > 37) than OQR varieties. Previous studies have indicated that starch with a higher proportion of short chains tends to have smaller lamellar distances (9.1–9.4 nm) [39], which contributes to higher peak viscosity, greater breakdown, and ultimately results in cooked rice with increased stickiness, reduced hardness, improved elasticity, and slower retrogradation. These findings align with the characteristics we observed in HQR varieties with higher proportion of short chains. Previous studies have found that short amylopectin chains (DP 6–12) have low molecular weights and contain few branch points, allowing water to penetrate more easily during heating [32,40]. This facilitates their leaching from starch granules into the surrounding water during cooking. The leached short-chain branches are rich in hydroxyl groups; upon cooling, these groups interact through hydrogen bonding to form a viscous, continuous hydrated gel layer on the surface of cooked rice grains, directly contributing to the sensation of stickiness during consumption. In contrast, long-chain amylopectin (DP ≥ 37) tends to form stable crystalline regions that restrict granule swelling and the leaching of materials, thereby increasing firmness and reducing the stickiness of the cooked rice [41].

### 3.5. Gelation and Hydration Properties of Starch

**Figure 3:**
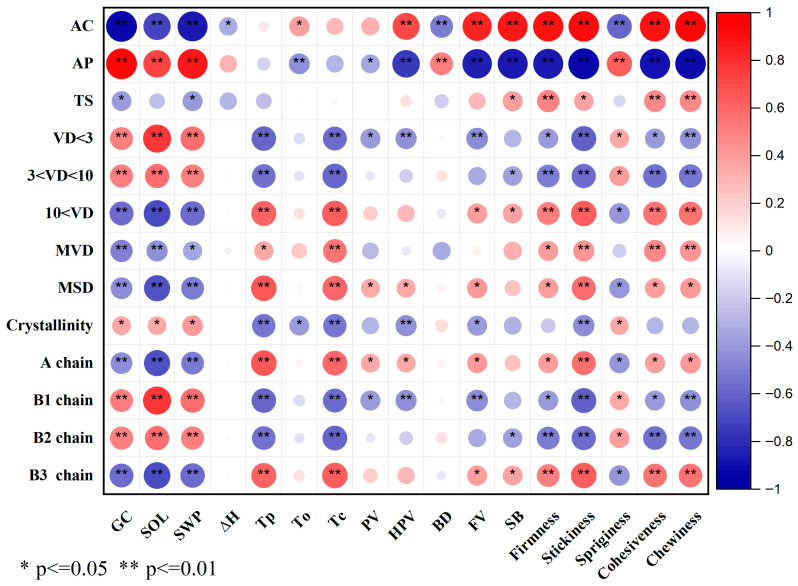
Correlation coefficient between cooked rice textural properties and starch components, structures and physicochemical properties across HQR and OQR varieties. Each correlation coefficient shows the average value of three replicates per correlation factor. * Correlation is significant at the 0.05 level, ** correlation is significant at the 0.01 level. AC, amylose content; AP, amylopectin content; TS, total starch content; VD < 3: small granules (<3 μm); 3 < VD < 10: medium granules (3–10 μm); VD > 10: large granules (>10 μm); MVD: mean diameter of volume; MSD: mean diameter of surface area; A chain: amylopectin chains with DP 6–12; B1 chain: amylopectin chains with DP 13–24; B2 chain: amylopectin chains with DP 25–36; B3 chain: amylopectin chains with DP > 36; GC: gel consistency; SOL: starch solubility; SWP: swelling power; △H: enthalpy of gelatinization; Tp: peak temperature; To: onset temperature; Tc: conclusion temperature; PV: peak viscosity; HPV: hot paste viscosity; BD: breakdown viscosity; FV: final viscosity; SB: setback viscosity.

The differences in gel consistency, swelling power, and water solubility of starch among the HQR and OQR varieties were statistically significant (Table 5). Specifically, HQR varieties typically demonstrated a longer gel consistency, as well as elevated starch solubility and swelling power when compared to the OQR types. The solubility of starch indicates the capacity of its molecules to disperse within water, a characteristic that is determined by both the molecular configuration and external circumstances [23]. Typically, the linear configuration of amylose diminishes solubility due to enhancing robust intermolecular interactions and the creation of a compact network [42]. In agreement with this, our findings revealed a notable negative correlation between the content of amylose and its solubility (Figure 3).

The swelling power, which reflects the capacity of starch granules to retain water, can be influenced by various factors, including molecular weight, the presence of lipid-amylose complexes, the ratio of amylose to amylopectin, and the structural organization of both amorphous and crystalline regions within the granules [23,43]. Our findings indicated that there is a negative correlation between swelling power and amylose content, as well as the amounts of A-chain and B3-chain of amylopectin, in addition to larger starch granules. In contrast, a positive correlation was observed with the levels of B1-chain and B2-chain of amylopectin, along with smaller granules. Generally, when starch is subjected to heating in a large volume of water, the disruption of hydrogen bonds occurs, resulting in the deterioration of crystalline structures. Subsequently, water molecules establish hydrogen bonds with the exposed hydroxyl groups of both amylose and amylopectin, facilitating granule swelling and enhancing solubility [35].

### 3.6. Starch Thermal Properties

The gelatinization characteristics, which indicate the ratio of crystallites associated with molecular arrangement, were described through ΔH (melting enthalpy), Tp (peak temperature), To (onset temperature), and Tc (conclusion temperature). As illustrated in Table 5, notable differences in these thermal characteristics were found between the HQR and OQR varieties. Although WXYSZ, YXYHS, and YXYLS presented significantly lower ΔH values, other HQR varieties displayed either significantly higher or comparable ΔH values compared to OQR varieties. Gelatinization enthalpy signifies the energy necessary to melt microcrystals and break double helices by disrupting inter- and intra-helical hydrogen bonds. An increased amylopectin content leads to a rise in ΔH, as it facilitates the formation of additional double helices and crystallites [44]. Nonetheless, our investigation revealed no link between ΔH and the content of amylopectin, nor its branch chain distribution (Figure 3).

The To values for HQR varieties were significantly lower or sat between the values noted for OQR varieties. Conversely, TFY208 and WXY982 exhibited significantly higher Tp and Tc values in comparison to OQR varieties, while other HQR varieties had notably reduced Tp and Tc values. Importantly, TFY208 and WXY982 contained greater amounts of B1 and B3 chains alongside lower levels of A and B2 chains. This finding supports the assertion of Zhang et al. [45], who noted that a high proportion of long-chain amylopectin is a critical factor leading to elevated gelatinization temperatures.

### 3.7. Starch Pasting Properties

RVA profiling is an essential method for assessing the eating quality of rice, as it replicates the cooking process of rice to determine the properties of starch pasting. Among HQR varieties, LJY2, TYXZ, TFY208 and TLY131 exhibited relatively higher peak viscosity, with values comparable to those of OQR varieties, whereas the other HQR varieties had significantly lower peak viscosity than the latter (Table 6). Meanwhile, markedly lower hot viscosity, final viscosity, and setback were observed in HQR varieties than in OQR varieties. On the contrary, the HQR varieties (excluding TYN39 and YXYLS) showed significantly higher breakdown than OQR varieties. Typically, rice with superior eating quality is closely associated with high breakdown and low setback values, which contributes to a soft post-cooking texture and enhanced palatability [6,46]. In the present study, the combination of higher breakdown (ranging from 1423.3 cP to 2556.3 cP) and lower setback (ranging from −1360.7 cP to 233.3 cP) in HQR varieties suggest that their cooked rice is less likely to harden or retrograde during cooking compared to OQR varieties.

### 3.8. Cooked Rice Texture Characteristics

The texture of cooked rice is generally evaluated based on firmness, stickiness, springiness, cohesiveness, and chewiness. These texture parameters varied significantly among HQR and OQR varieties (Table 7). Compared with OQR varieties, HQR varieties consistently showed significantly lower values in firmness, cohesiveness, and chewiness, but exhibited higher springiness and greater absolute stickiness values. These results align with the reported preference in East Asia for rice that is chewy, sticky, and relatively soft [6].

Amylose content is well established as a key determinant of rice firmness, showing a significant positive correlation, which is consistent with our findings (Figure 3). The stickiness of rice is mainly affected by the leaching of amylose and amylopectin during the cooking process. Meanwhile, the decrease in firmness and the emergence of stickiness are largely influenced by the molecular mobility of starch components [24]. In this study, a highly significant negative correlation was observed between both firmness and stickiness with starch solubility and swelling power. While Li et al. [47] indicated that an increased content and longer chain lengths of amylopectin lead to a tougher and less sticky texture, our findings revealed positive correlations between firmness and stickiness with the proportions of A chains and B3 chains of amylopectin, contrasted with negative correlations involving B1 and B2 chains. Additionally, cohesiveness, firmness, stickiness, and chewiness were found to be negatively correlated with the ratios of small and medium-sized starch granules, whereas a positive correlation was noticed with larger granules. This phenomenon may result from the elevated ratio of larger granules enhancing stronger intermolecular interactions during cooking, thus necessitating greater force to separate the rice grains.

### 3.9. The Relationship Between the Physicochemical Properties of Starch and Eating Quality

**Figure 4:**
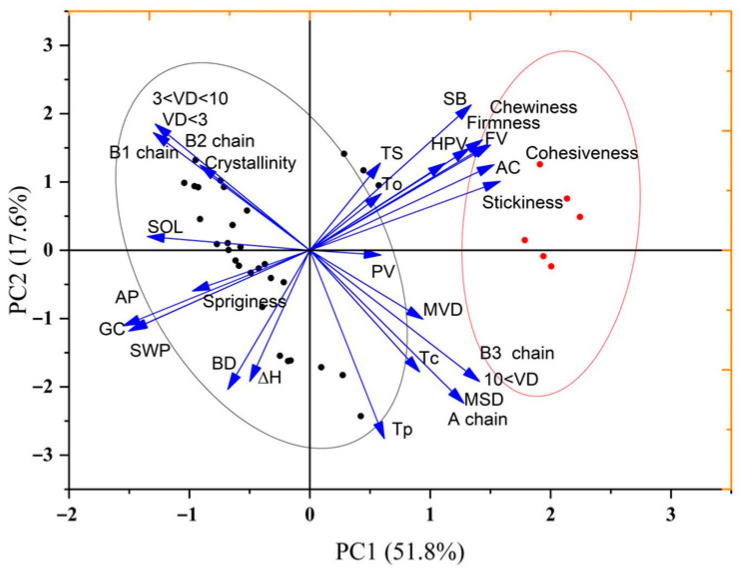
Principal component analysis between cooked rice textural properties and starch components, structures and physicochemical properties across HQR and OQR varieties. The black dots represent HQR, and the red dots represent OQR. The black and red ellipses represent the 95% confidence ellipses for HQR and OQR, respectively. The arrows indicate the factor loadings. AC, amylose content; AP, amylopectin content; TS, total starch content; VD < 3: small granules (<3 μm); 3 < VD < 10: medium granules (3–10 μm); VD > 10: large granules (>10 μm); MVD: mean diameter of volume; MSD: mean diameter of surface area; A chain: amylopectin chains with DP 6–12; B1 chain: amylopectin chains with DP 13–24; B2 chain: amylopectin chains with DP 25–36; B3 chain: amylopectin chains with DP > 36; GC: gel consistency; SOL: starch solubility; SWP: swelling power; △H: enthalpy of gelatinization; Tp: peak temperature; To: onset temperature; Tc: conclusion temperature; PV: peak viscosity; HPV: hot paste viscosity; BD: breakdown viscosity; FV: final viscosity; SB: setback viscosity.

Principal component analysis (Figure 4) revealed that high-quality rice (HQR) clustered on the positive side of PC1, which was associated with A chains, B1 chains, crystallinity, gelatinization temperatures (Tp, To, Tc), and enthalpy (ΔH). In contrast, low-quality rice was located on the negative side of PC1 and correlated with B3 chains, large granules (>10 μm), high setback (SB), and elevated hardness. According to Amagliani et al. [48], A and B1 chains form double helices in crystalline regions. Their higher proportions enhance crystallinity and gelatinization temperatures, consistent with the positive correlations observed. Conversely, excessive-length B3 chains (>DP 36) impede crystal formation, correlating with their association with low-quality rice, high SB, and hardness [41]. In addition, high amylose content was associated with firmer texture and higher setback, while short chains and small granules (<3 μm) exhibited negative correlations with swelling power and solubility, contributing to a softer texture. In summary, high-quality rice is characterized by moderate amylose content (14–20%), elevated A and B1 chains, low B3 chains, high crystallinity, high gelatinization temperatures, and low setback, resulting in soft, non-sticky, and cooling-resistant cooked rice. In contrast, low-quality rice displays extreme amylose levels, increased B3 chains, reduced crystallinity, heightened setback, and greater hardness, leading to a rough texture and rapid staling.

## 4. Conclusions

This study provides a comprehensive analysis of the multiscale structural and physicochemical characteristics of HQR and OQR varieties, with a focus on starch granule distribution, crystalline structure, and chain length distribution of amylopectin, as well as functional properties including pasting, thermal, and textural properties. The results indicate that HQR varieties exhibit a lower amylose content (16.6–20.2%), which shows a strong positive correlation with hot viscosity, final viscosity, setback, firmness, stickiness, cohesiveness, and chewiness, but a negative correlation with breakdown and springiness. Compared with OQR varieties, HQR varieties contain a higher proportion of short chains and fewer long chains in amylopectin, along with a relatively greater abundance of small- and medium-sized granules and fewer large granules. Furthermore, HQR varieties demonstrate lower hot viscosity, final viscosity, setback, and hardness, but higher breakdown, stickiness, and springiness than OQR varieties. These findings will support breeding efforts that target amylose content, amylopectin chain length, and granule size distribution as key selection traits. Industrially, HQR varieties are suitable for soft-textured products, whereas OQR varieties better meet the requirements of applications demanding structural firmness. Future work should explore the molecular mechanisms underlying these traits, their environmental stability, and their implications for digestibility and nutritional quality.

## Figures and Tables

**Table 1:** Details of the tested HQR and OQR varieties.

Type	Variety	Sterile Line	Restorer Line	Released Year	The Year it Received the National High-Quality Rice Gold Award
HQR	TLY131	T764S	R131	2020	2020
	LJY2	Longjing4302A	Huahui3621	2016	2019
	TFY208	TaifengA	Guanghui208	2012	2019, 2020, 2023
	TY553	TaifengA	R553	2019	2019
	TYN39	TaifengA	Nongxiang39	2021	2024
	TYXZ	Taonong1A	Huanghuazhan	2015	2018
	WXY982	WangxiangA	HongR982	2019	2019, 2020
	WXYSZ	WangxiangA	Shuangzhan	2017	2020, 2023, 2024, 2025
	YXYHS	YexiangA	RHaisi	2019	2018
	YXYLS	YexiangA	RLisi	2017	2020
OQR	JYT025	JifengA	CanghuiT025	2017	
	JYH1573	JifengA	Yuehuihang1573	2015	

**Table 2:** Starch components and protein content of HQR and OQR varieties.

Type	Variety	Amylose Content (%)	Amylopectin Content (%)	Total Starch Content (%)	Protein Content (%)
HQR	TLY131	17.5 ± 0.4 de	64.5 ± 0.3 ab	82.0 ± 0.6 a	6.3 ± 0.0 f
	LJY2	17.8 ± 1.0 d	64.1 ± 0.5 b	82.0 ± 0.5 a	6.4 ± 0.1 ef
	TFY208	17.6 ± 0.3 d	64.4 ± 0.4 b	82.0 ± 0.3 a	6.7 ± 0.1 bc
	TY553	17.7 ± 0.7 d	64.4 ± 0.3 ab	82.1 ± 0.5 a	6.0 ± 0.0 g
	TYN39	17.9 ± 0.6 d	64.6 ± 0.1 ab	82.5 ± 0.7 a	6.5 ± 0.0 de
	TYXZ	17.9 ± 0.6 d	64.2 ± 0.3 b	82.1 ± 0.5 a	6.3 ± 0.0 f
	WXY982	16.6 ± 0.7 e	65.0 ± 0.4 a	81.6 ± 0.4 a	7.3 ± 0.1 a
	WXYSZ	20.2 ± 0.7 c	62.6 ± 0.4 c	83.1 ± 0.5 a	6.6 ± 0.1 cd
	YXYHS	17.1 ± 0.6 de	65.0 ± 0.3 a	82.1 ± 0.5 a	6.4 ± 0.0 ef
	YXYLS	17.4 ± 0.6 de	64.2 ± 0.3 b	81.6 ± 0.4 a	6.8 ± 0.2 b
OQR	JYT025	26.1 ± 0.5 a	56.4 ± 0.6 e	82.5 ± 0.2 a	6.8 ± 0.2 b
	JYH1573	22.6 ± 0.6 b	59.6 ± 0.4 d	82.3 ± 0.1 a	5.8 ± 0.1 h
ANOVA	Variety	74.1 **	177.9 **	2.0 ^ns^	48.6 **
	Type	154.2 **	206.9 **	3.6 ^ns^	1.6 ^ns^

Data are expressed as the mean ± standard deviation; values with different lowercase letters in the same column indicate a statistically significant difference (*p* < 0.05) between two varieties. ^ns^ denotes no significant difference at the 0.05 probability level; ** indicates significant differences at the 0.01 probability levels.

**Table 3:** Starch particle size distribution and degree of crystallinity of HQR and OQR varieties.

Type	Variety	Small Starch Granules < 3 μm (%)	Medium Starch Granules 3–10 μm (%)	Large Starch Granules > 10 μm (%)	Mean Diameter of Volume (μm)	Mean Diameter of Surface Area (μm)	Degree of Crystallinity (%)
HQR	TLY131	12.0 ± 0.9 a	60.5 ± 1.8 de	27.5 ± 2.7 f	16.9 ± 1.6 d	4.9 ± 0.3 g	30.9 ± 0.5 bc
	LJY2	8.7 ± 0.9 de	61.0 ± 1.5 cd	30.2 ± 2.1 de	12.8 ± 0.7 f	5.7 ± 0.2 e	27.8 ± 0.7 ef
	TFY208	7.1 ± 0.7 fg	53.4 ± 1.0 gh	39.5 ± 1.2 ab	20.3 ± 1.8 ab	6.7 ± 0.5 ab	26.4 ± 1.8 fg
	TY553	9.1 ± 0.6 cde	59.4 ± 1.9 de	31.5 ± 1.8 cd	18.1 ± 1.4 cd	5.7 ± 0.1 e	26.2 ± 0.5 fgh
	TYN39	10.1 ± 0.7 bc	57.4 ± 1.2 f	32.5 ± 1.9 cd	21.5 ± 1.1 a	5.4 ± 0.5 ef	32.8 ± 2.2 a
	TYXZ	8.1 ± 0.4 ef	63.3 ± 1.9 ab	28.6 ± 1.6 ef	12.4 ± 1.0 f	5.8 ± 0.2 de	32.3 ± 0.5 ab
	WXY982	7.1 ± 0.4 fg	59.3 ± 0.3 def	33.6 ± 0.5 c	17.8 ± 0.4 cd	6.2 ± 0.2 cd	24.5 ± 0.6 h
	WXYSZ	9.6 ± 0.2 cd	59.1 ± 1.5 ef	31.3 ± 1.7 cd	20.9 ± 0.8 a	5.4 ± 0.3 ef	28.8 ± 0.9 de
	YXYHS	10.8 ± 0.4 b	65.1 ± 1.0 a	24.1 ± 0.8 g	9.8 ± 0.4 g	5.0 ± 0.1 fg	29.7 ± 0.5 cd
	YXYLS	10.8 ± 0.8 b	62.5 ± 1.5 bc	26.7 ± 1.7 f	15.0 ± 1.1 e	5.0 ± 0.2 fg	26.7 ± 0.7 fg
OQR	JYT025	6.4 ± 0.7 gh	55.2 ± 0.7 g	38.4 ± 1.3 b	21.0 ± 1.7 a	6.5 ± 0.5 bc	25.8 ± 0.5 gh
	JYH1573	6.0 ± 0.6 h	52.2 ± 1.1 h	41.7 ± 1.3 a	18.9 ± 1.1 bc	7.0 ± 0.3 a	25.1 ± 0.6 gh
ANOVA	Variety	27.3 **	36.1 **	42.2 **	50.9 **	20.3 **	23.1 **
	Type	19.5 **	19.6 **	26.1 **	4.2 *	19.7 **	6.8 *

Data are expressed as the mean ± standard deviation; values with different lowercase letters in the same column indicate a statistically significant difference (*p* < 0.05) between two varieties. * and ** indicate significant differences at the 0.05 and 0.01 probability levels, respectively.

**Table 4:** Chain length distribution of amylopectin of HQR and OQR varieties.

Type	Variety	A (DP 6–12) (%)	B1 (DP 13–24) (%)	B2 (DP 25–36) (%)	B3 (Chain DP > 37) (%)
HQR	TLY131	29.0 ± 0.7 ab	49.3 ± 0.3 d	11.3 ± 0.2 b	10.4 ± 0.2 de
	LJY2	28.9 ± 0.5 ab	49.2 ± 0.3 d	11.3 ± 0.1 b	10.6 ± 0.1 cde
	TFY208	25.4 ± 0.4 c	52.9 ± 0.2 b	11.0 ± 0.1 c	10.7 ± 0.1 bcd
	TY553	29.0 ± 0.2 ab	49.1 ± 0.0 d	11.3 ± 0.1 b	10.6 ± 0.1 cde
	TYN39	28.9 ± 0.5 ab	49.5 ± 0.3 d	11.1 ± 0.1 bc	10.5 ± 0.1 de
	TYXZ	29.0 ± 0.2 ab	49.1 ± 0.0 d	11.3 ± 0.1 b	10.7 ± 0.1 abcd
	WXY982	23.6 ± 0.2 d	54.4 ± 0.8 a	11.0 ± 0.4 c	11.0 ± 0.6 ab
	WXYSZ	25.7 ± 1.0 c	52.6 ± 0.7 bc	11.0 ± 0.1 c	10.7 ± 0.2 abcd
	YXYHS	28.6 ± 1.0 b	49.2 ± 0.2 d	11.6 ± 0.5 a	10.6 ± 0.5 cde
	YXYLS	29.4 ± 0.9 a	48.9 ± 0.6 d	11.3 ± 0.1 b	10.3 ± 0.2 e
OQR	JYT025	26.0 ± 1.0 c	52.1 ± 1.1 c	11.0 ± 0.2 c	10.9 ± 0.2 abc
	JYH1573	25.6 ± 0.5 c	52.3 ± 0.3 bc	11.0 ± 0.1 c	11.0 ± 0.1 a
ANOVA	Variety	56.0 **	47.8 **	6.0 **	3.4 **
	Type	5.0 *	4.2 *	4.3 *	10.3 **

Data are presented as the mean ± standard deviation; values with different lowercase letters in the same column indicate a statistically significant difference (*p* < 0.05) between two varieties. * and ** indicate significant differences at the 0.05 and 0.01 probability levels, respectively.

**Table 5:** Gel consistency, starch solubility and swelling power, and thermal properties of HQR and OQR varieties.

Type	Variety	Gel Consistency (mm)	Starch Solubility (%)	Swelling Power (g/g)	∆H (J/g)	To (°C)	Tp (°C)	Tc (°C)
HQR	TLY131	74.8 ± 1.3 cde	18.2 ± 1.4 a	23.3 ± 0.4 ab	5.0 ± 0.1 a	54.9 ± 1.7 gh	70.5 ± 1.2 d	78.1 ± 1.6 d
	LJY2	83.7 ± 1.5 a	15.4 ± 0.4 bc	22.1 ± 0.9 cd	4.6 ± 0.1 b	57.5 ± 1.0 def	68.2 ± 1.4 e	76.1 ± 0.2 e
	TFY208	73.7 ± 1.5 ef	15.2 ± 0.6 bc	24.0 ± 0.4 a	4.3 ± 0.2 c	55.8 ± 0.4 fg	77.0 ± 1.3 b	83.5 ± 1.4 b
	TY553	67.3 ± 2.5 g	16.0 ± 0.6 b	21.8 ± 0.8 d	4.9 ± 0.2 a	55.7 ± 1.0 fg	69.2 ± 0.3 de	76.8 ± 0.4 de
	TYN39	77.7 ± 1.5 bc	15.7 ± 0.3 bc	23.3 ± 0.6 ab	4.5 ± 0.1 b	59.0 ± 1.5 cde	70.9 ± 1.4 d	77.5 ± 1.3 de
	TYXZ	71.2 ± 3.0 f	15.3 ± 0.5 bc	22.3 ± 0.5 cd	4.6 ± 0.2 b	53.0 ± 1.9 h	68.3 ± 1.7 e	76.2 ± 0.7 e
	WXY982	77.3 ± 2.1 bcd	16.0 ± 0.3 b	22.2 ± 1.0 cd	5.0 ± 0.1 a	61.9 ± 1.5 b	79.2 ± 1.0 a	85.5 ± 1.6 a
	WXYSZ	61.0 ± 3.1 h	14.8 ± 0.4 cd	17.7 ± 0.2 e	3.6 ± 0.1 f	59.6 ± 0.9 cd	65.8 ± 0.7 f	82.1 ± 2.3 bc
	YXYHS	78.7 ± 2.3 b	17.6 ± 0.3 a	23.0 ± 0.3 bc	4.0 ± 0.1 d	59.8 ± 2.0 bc	69.3 ± 1.8 de	77.5 ± 0.8 de
	YXYLS	74.3 ± 2.1 def	16.1 ± 0.4 b	22.5 ± 1.0 bcd	4.0 ± 0.1 d	61.1 ± 2.1 bc	68.8 ± 1.0 e	76.4 ± 0.3 e
OQR	JYT025	35.0 ± 1.0 j	13.1 ± 0.4 e	15.2 ± 0.7 f	4.2 ± 0.1 c	64.9 ± 1.0 a	74.3 ± 1.1 c	81.8 ± 0.6 c
	JYH1573	52.8 ± 2.0 i	13.9 ± 0.8 de	13.3 ± 0.9 g	4.2 ± 0.1 c	57.0 ± 1.1 efg	73.3 ± 0.6 c	81.2 ± 0.2 c
ANOVA	Variety	144.6 **	14.0 **	116.1 **	41.3 **	48.7 **	19.2 **	31.4 **
	Type	87.7 **	25.6 **	110.8 **	1.2 ^ns^	4.1 *	4.3 *	3.7 ^ns^

∆H = gelatinization enthalpy; To = onset temperature; Tp = peak gelatinization temperature; Tc = conclusion temperature. Data are expressed as the mean ± standard deviation; values with different lowercase letters in the same column indicate a statistically significant difference (*p* < 0.05) between two varieties. ^ns^ denotes no significant difference at the 0.05 probability level; * and ** indicate significant differences at the 0.05 and 0.01 probability levels, respectively.

**Table 6:** Starch pasting properties of HQR and OQR varieties.

Type	Variety	Peak Viscosity (cP)	Hot Viscosity (cP)	Breakdown (cP)	Final Viscosity (cP)	Setback (cP)
HQR	TLY131	4084.7 ± 53.4 c	2013.7 ± 37.2 d	2071.0 ± 32.4 c	3360.3 ± 37.4 d	−724.3 ± 53.6 f
	LJY2	4391.7 ± 93.3 a	2118.0 ± 69.0 c	2273.7 ± 93.8 b	3378.0 ± 69.4 d	−1013.7 ± 30.2 g
	TFY208	4254.0 ± 64.4 b	1697.7 ± 25.5 ef	2556.3 ± 73.7 a	2893.3 ± 81.6 e	−1360.7 ± 33.0 h
	TY553	3683.3 ± 96.5 e	1778.7 ± 82.3 e	1904.7 ± 23.2 d	2899.0 ± 55.5 e	−784.3 ± 82.9 f
	TYN39	2121.0 ± 59.1 h	609.0 ± 38.7 g	1512.0 ± 25.6 f	1346.0 ± 58.0 g	−775.0 ± 23.8 f
	TYXZ	4318.7 ± 80.5 ab	2034.7 ± 52.2 cd	2284.0 ± 30.5 b	3288.0 ± 46.6 d	−1030.7 ± 34.2 g
	WXY982	3523.7 ± 87.8 f	1664.0 ± 77.1 f	1859.7 ± 15.5 d	2548.7 ± 100.0 f	−975.0 ± 38.5 g
	WXYSZ	3721.7 ± 66.7 de	2054.0 ± 79.5 cd	1667.7 ± 31.7 e	3955.0 ± 102.3 c	233.3 ± 44.4 c
	YXYHS	3825.3 ± 39.5 d	1979.0 ± 36.0 d	1846.3 ± 70.7 d	3319.7 ± 91.9 d	−505.7 ± 78.2 e
	YXYLS	3164.3 ± 85.4 g	1741.0 ± 19.0 ef	1423.3 ± 68.1 gh	2910.3 ± 65.0 e	−254.0 ± 22.5 d
OQR	JYT025	4385.3 ± 85.7 a	3018.7 ± 89.7 a	1366.7 ± 17.2 h	5455.3 ± 116.2 a	1070.0 ± 61.5 a
	JYH1573	4109.0 ± 69.3 c	2620.7 ± 39.1 b	1488.3 ± 39.0 fg	4994.7 ± 89.0 b	885.7 ± 19.7 b
ANOVA	Variety	233.0 **	282.9 **	245.9 **	562.6 **	748.0 **
	Type	3.7 ^ns^	31.9 **	11.8 **	59.3 **	82.0 **

Data are expressed as the mean ± standard deviation; values with different lowercase letters in the same column indicate a statistically significant difference (*p* < 0.05) between two varieties. ^ns^ denotes no significant difference at the 0.05 probability level; ** indicates significant differences at the 0.01 probability levels.

**Table 7:** Rice texture characteristics of HQR and OQR varieties.

Type	Cultivar	Firmness (g)	Stickiness (g)	Springiness	Cohesiveness	Chewiness
HQR	TLY131	2422.6 ± 40.2 e	−471.4 ± 3.8 gh	0.73 ± 0.04 bc	0.35 ± 0.01 d	854.6 ± 26.7 de
	LJY2	2218.2 ± 78.2 g	−456.3 ± 11.0 fg	0.72 ± 0.03 bc	0.30 ± 0.01 gh	629.0 ± 25.9 g
	TFY208	2424.9 ± 72.4 e	−434.2 ± 18.1 e	0.75 ± 0.02 ab	0.34 ± 0.00 de	829.9 ± 39.4 e
	TY553	1963.4 ± 71.4 h	−385.3 ± 14.5 c	0.66 ± 0.03 de	0.31 ± 0.00 fg	627.0 ± 41.9 g
	TYN39	2287.5 ± 11.9 fg	−476.1 ± 5.0 gh	0.71 ± 0.01 bc	0.33 ± 0.01 ef	752.3 ± 14.9 f
	TYXZ	2304.1 ± 53.9 f	−409.6 ± 21.4 d	0.74 ± 0.03 abc	0.31 ± 0.00 g	723.7 ± 31.2 f
	WXY982	2011.3 ± 30.0 h	−449.2 ± 20.8 ef	0.76 ± 0.03 ab	0.29 ± 0.01 h	587.4 ± 35.0 g
	WXYSZ	3361.6 ± 62.4 c	−314.2 ± 8.1 b	0.78 ± 0.01 a	0.47 ± 0.0 c	1557.2 ± 39.2 c
	YXYHS	2551.0 ± 8.6 d	−482.8 ± 9.7 h	0.75 ± 0.04 ab	0.35 ± 0.01 de	894.4 ± 12.2 d
	YXYLS	2245.7 ± 24.2 fg	−408.4 ± 5.4 d	0.69 ± 0.04 cd	0.33 ± 0.00 de	754.3 ± 20.8 f
OQR	JYT025	3751.3 ± 74.1 a	−167.0 ± 5.5 a	0.62 ± 0.04 ef	0.53 ± 0.02 a	2052.1 ± 72.1 a
	JYH1573	3587.8 ± 31.9 b	−180.0 ± 7.5 a	0.60 ± 0.00 f	0.51 ± 0.01 b	1834.1 ± 14.1 b
ANOVA	Variety	510.7 **	226.7 **	9.7 **	209.8 **	8.7 **
	Type	63.7 **	137.6 **	38.1 **	72.1 **	92.2 **

Data are expressed as the mean ± standard deviation; values with different lowercase letters in the same column indicate a statistically significant difference (*p* < 0.05) between two varieties. ** indicates significant differences at the 0.01 probability levels.

## Data Availability

The original contributions presented in this study are included in the article. Further inquiries can be directed to the corresponding author.
